# Data-driven analysis of a validated risk score for ovarian cancer identifies clinically distinct patterns during follow-up and treatment

**DOI:** 10.1038/s43856-022-00193-6

**Published:** 2022-10-01

**Authors:** Stefan Enroth, Emma Ivansson, Julia Hedlund Lindberg, Maria Lycke, Jessica Bergman, Anna Reneland, Karin Stålberg, Karin Sundfeldt, Ulf Gyllensten

**Affiliations:** 1 Department of Immunology, Genetics, and Pathology, Biomedical Center, SciLifeLab Uppsala, Uppsala University, SE-75108 Uppsala, Sweden; 2 Swedish Collegium for Advanced Study, Thunbergsvägen 2, SE-752 38 Uppsala, Sweden; 3 Department of Obstetrics and Gynecology, Institute of Clinical Sciences, Sahlgrenska Academy at Gothenburg University, SE-416 85 Gothenburg, Sweden; 4 Olink Proteomics AB, Uppsala, Sweden; 5 Department of Women’s and Children’s Health, Uppsala University, SE-751 85 Uppsala, Sweden

**Keywords:** Ovarian cancer, Diagnostic markers, Prognostic markers

## Abstract

**Background:**

Ovarian cancer is the eighth most common cancer among women and due to late detection prognosis is poor with an overall 5-year survival of 30–50%. Novel biomarkers are needed to reduce diagnostic surgery and enable detection of early-stage cancer by population screening. We have previously developed a risk score based on an 11-biomarker plasma protein assay to distinguish benign tumors (cysts) from malignant ovarian cancer in women with adnexal ovarian mass.

**Methods:**

Protein concentrations of 11 proteins were characterized in plasma from 1120 clinical samples with a custom version of the proximity extension assay. The performance of the assay was evaluated in terms of prediction accuracy based on receiver operating characteristics (ROC) and multiple hypothesis adjusted Fisher’s Exact tests on achieved sensitivity and specificity.

**Results:**

The assay’s performance is validated in two independent clinical cohorts with a sensitivity of 0.83/0.91 and specificity of 0.88/0.92. We also show that the risk score follows the clinical development and is reduced upon treatment, and increased with relapse and cancer progression. Data-driven modeling of the risk score patterns during a 2-year follow-up after diagnosis identifies four separate risk score trajectories linked to clinical development and survival. A Cox proportional hazard regression analysis of 5-year survival shows that at time of diagnosis the risk score is the second-strongest predictive variable for survival after tumor stage, whereas MUCIN-16 (CA-125) alone is not significantly predictive.

**Conclusion:**

The robust performance of the biomarker assay across clinical cohorts and the correlation with clinical development indicates its usefulness both in the diagnostic work-up of women with adnexal ovarian mass and for predicting their clinical course.

## Plain language summary

Ovarian cancer is commonly detected at a late stage, resulting in poor long-term outcomes with only 30–50% of women surviving more than 5 years after diagnosis. New ways to accurately detect ovarian cancer are needed to increase survival rates. We have previously developed a score based on the levels of certain proteins in the blood, which can be used to detect ovarian cancer. Here, we confirm that this score allows us to distinguish ovarian cancer from benign cysts. We also show that the score is reduced upon cancer treatment and increases with disease recurrence, and that it is predictive of patient survival. A simple blood test such as this would be useful both for detection of disease and for follow-up during treatment, and might be an important tool to help improve outcomes for patients with ovarian cancer.

## Abstract (web summary)

Enroth et al. validate their previously developed 11-biomarker plasma protein-based risk-score for distinguishing benign vs. malignant ovarian tumors. The risk score is found to be informative of treatment outcome and predictive of 5-year survival.

## Introduction

Ovarian cancer is currently the eighth most common cancer among women across the world, with over 300,000 cases and 200,000 deaths per year, and an estimated global incidence of 6.6 per 100,000 women per year^1^. Detection of the cancer is usually late with less than one-third of cases discovered in stage I or II, resulting in poor prognosis with an overall 5-year survival rate of only 30–50%^2^. The overall 5-year survival rate varies greatly depending on tumor stage at diagnosis, and it is close to 90% when the tumor is detected in stage I, but only 20% for stage IV^2^. The precursor states of ovarian cancers have proven difficult to identify. Precise knowledge of the etiology of the cancer could help determine an optimized screening interval in relation to cancer development. However, it has been suggested that serous tubal intraepithelial carcinomas (STIC), the presumed precursor to ovarian high-grade serous carcinomas, develop slowly with up to two decades from the first occurrence of genetic predisposing mutations^3^. Recent molecular evidence from patient material suggests that the developing of ovarian cancer from STIC can occur in a much shorter time, across an estimated timespan of 6–7 years^4,5^. Additional estimates based on tumor size and growth^6^ indicate that ovarian cancer can spend over 4 years in situ, or as stage I and II, before progressing to stages III and IV. Today discovery is mainly symptom-driven and women who experience pelvic symptoms are typically examined with transvaginal ultrasound (TVU) or computer tomography, and when these indicate an adnexal ovarian mass, surgery provides the final diagnosis. However, a majority of patients undergoing surgery actually have benign cysts, and more effective and targeted preoperative tools to predict malignancy would reduce unnecessary operations and minimize potential complications and induced premature menopause.

Available biomarkers for ovarian cancer such as MUCIN-16 (CA-125) or WAP Four-Disulfide Core Domain 2 (WFDC2 or HE4) are used as a complement to imaging examinations. MUCIN-16 was introduced as a biomarker for ovarian cancer in 1983^7^ and is currently the most important single biomarker for diagnosis and management of ovarian cancer^8^. However, MUCIN-16 alone has low sensitivity for early-stage cancer partly due to the large proportion of false positives linked to relatively benign gynecological conditions such as endometriosis, infections, or pregnancies^8^. Combinations of CA-125 with other biomarkers, including WFDC2, such as in the ROMA Score (Ovarian Malignancy Risk Algorithm), can achieve a sensitivity of up to 75% at a specificity of 90–95%^9,10^. But again, the low sensitivity for detection of early-stage ovarian cancer (stages I and II), and the resulting high cost and risk of over-treatment, prohibits population screening using these biomarkers. Previous studies predicting the risk of malignancy in adnexal ovarian mass using only TVU^11^ report sensitivities ranging from 99.7 to 89.0%, with specificities of 33.7 to 84.7%; thus TVU in the hands of specialists can out-perform molecular tests^12^. However, these highly specialized units are scarce, whereas a molecular test could be objectively performed without the need for highly trained experts.

We have previously developed a risk score for separating benign from malignant tumors based on analysis of eleven (11) plasma proteins (MUCIN-16, SPINT1, TACSTD2, CLEC6A, ICOSLG, MSMB, PROK1, CDH3, WFDC2, KRT19, and FR-alpha) plus age^13^. Previously, we used one discovery cohort and two independent validation cohorts to select proteins and evaluated the performance of the models using relative protein concentrations as reported by the proximity extension assay (PEA)^14^. Based on separate validation cohorts the risk score model was finalized with fixed coefficients based on measurements in absolute concentrations^13^. In the previously studied cohort, we achieved a sensitivity of 0.85 and a specificity of 0.93 in separating ovarian cancer tumors in stages I–IV from benign tumors.

In the present study, we validate the performance of the multiplex protein assay in two independent Swedish patient cohorts at time of diagnosis. We also analyze serially collected samples from one of these cohorts to study the development of the risk score during treatment and follow-up of ovarian, endometrial and cervical cancer. The risk score is also analyzed in samples collected from healthy women in a third, cross-sectional Swedish cohort. We show that the performance of multiplex protein assay is robust and that the risk score pattern after diagnosis follows common clinical responses during treatment and relapse/progression and may therefore also be useful in monitoring clinical developments during follow-up.

## Methods

### Clinical cohorts

The samples were from three separate cohorts: the Biomovca cohort^15^, the UCAN biobank^16^, and the Northern Swedish Population Health Study (NSPHS)^17^. NSPHS is a population-based health study^17^ from which 87 healthy age-matched controls were selected. These samples were collected in 2006. The Biomovca is a clinical multicenter prospective cohort with samples from five secondary care centers and one tertiary care center in the region of Western Sweden^15^ and contains samples collected at time of diagnosis. These samples were collected between 2013 and 2016. A detailed clinical description of the characteristics of this cohort has been published before^15^. In the current study, a total of 610 Biomovca samples (Table 1) were analyzed. The UCAN (Uppsala Cancer Cohort) is a prospective cohort collected in the Uppsala-Örebro region consisting of women who were treated at the Akademiska Sjukhuset, Uppsala, Sweden. The UCAN cohort includes samples collected from women at time of diagnosis, as well as serial samples from the same women collected during follow-up and treatment (Table 1). These samples were collected in 2012–2018. Inclusion criteria on diagnoses were epithelial ovarian cancer, fallopian tube cancer, and peritoneal cancer. At diagnosis, the samples from UCAN were characterized as high-grade serous carcinomas (HGSC, 54%), low-grade serous carcinomas (LGSC, 11%), endometroid carcinomas (12%), clear cell carcinomas (5%), combined clear cell and endometroid carcinomas (1%), mucinous carcinomas (3%), non-epithelial ovarian cancer (6%) and carcinosarcomas (5%). Two (2) percent of the samples had no histologic annotation available. The UCAN samples were assigned any of four groups depending on the clinical timepoint. Samples denoted “Primary” were collected when the tumor had been diagnosed but prior to commencing treatment. The category “Treatment ongoing” included samples collected from the commencing of surgery or chemotherapy until the end of the course of treatment. A common treatment span is typically six months, but this could be shorter or longer for individual cases. The category “Response to treatment” included samples collected during follow-up after completed treatment, and comprised women with partial or complete remission, and consequently decreased tumor burden. The fourth category, “Relapse/progression” included samples collected either when cancer was recurring after an initial positive response to treatment or when cancer was progressing. These two types were combined into one group, since they represent increasing tumor burden. Clinical follow-up was limited to 5 years after initial diagnosis. A proportion (*N* = 48, 40%) of the UCAN biobank samples collected at time of diagnosis were used in the 2nd replication cohort in Enroth et al.^13^ (Table 1). These overlapping samples were not used to select the proteins in the model nor to establish the model-coefficients or cut-off for malignancy. The overlapping samples have not been previously analyzed using the quantitative proximity extension assay (PEA) used here.

**Table 1:** Characteristics of clinical samples.

Cohort	Diagnose	Type^a^	N^b^	Age^d^	BMI^d^	OC Stage^c^				MUC16^d^	WFDC2^d^
						I	II	III	IV	(U/ml)	(pg/ml)
NSPHS											
	Healthy	n.a.	87	63.7 (10.9)	28.3 (5.1)					n.r.	n.r.
Biomovca											
	Benign	Prim	443 (5)	50.7 (15.5)	25.9 (4.7)					54.7 (163.9)	64.6 (43.4)
	Borderl.	Prim	31 (1)	55.6 (14.6)	25.6 (4.1)	29	1	1		195.5 (459.7)	79.8 (36.6)
	Ovarian	Prim	136 (1)	62.4 (12.0)	27.9 (14.6)	37	16	68	15	972.1 (1477.3)	528.8 (512.5)
UCAN											
	Benign	Prim	55 (3)	57.5 (14.4)	27.0 (5.2)					69.0	n.r
	Ovarian	Prim	66 (2)	61.5 (13.3)	25.7 (7.3)	15 (8)	7 (4)	22 (11)	22 (12)	1216.9	n.r.
	Ovarian	Resp	66 (1)	62.4 (10.5)	25.4 (7.5)	18 (2)	4	26	18	17.6	n.r.
	Ovarian	Rela	33 (1)	70.4 (11.6)	27.2 (8.6)	1	1	16 (2)	15	259.0	n.r.
	Ovarian	Treat	108	60.1 (12.3)	25.4 (12.2)	17 (2)	9 (1)	45 (2)	37 (3)	348.9	n.r
	Cervix	Prim	9	54.2 (9.6)	n.r.					n.r.	n.r.
	Cervix	Treat	28	53.3 (10.7)	n.r.					n.r.	n.r
	Cervix	After	12	57.6 (12.8)	n.r.					n.r.	n.r
	Endo	Prim	19 (1)	73.2 (11.1)	n.r.					n.r.	n.r.
	Endo	Treat	8 (1)	71.7 (10.7)	n.r.					n.r.	n.r
	Endo	After	18	71.2 (11.3)	n.r.					n.r.	n.r.
	Endo	Rela	1	83.5	n.r.					n.r.	n.r
			1120 (16)								

*n.a.* not applicable, *n.r.* not recorded.

^a^See “Methods” for full description of these categories. Prim—primary, Resp—responding to treatment, Rela—progress/relapse, Treat—ongoing treatment, After—after treatment was completed.

^b^Number in parenthesis indicate samples that were excluded upon quality control (“Methods”).

^c^Number in parenthesis indicate samples that were included in Enroth et al. as 2nd replication cohort.

^d^Indicated number is group mean (sd).

To study the cancer specificity of the risk score we also included samples from the UCAN biobank collected at time of diagnosis and at one follow-up occasion from 25 women (2 samples from each woman) diagnosed with invasive cervical cancer and 25 women (2 samples from each woman) diagnosed with endometrial cancer (Table 1). The 25 cases with cervical cancer included the following diagnoses: squamous cell carcinomas (60.0%), adenocarcinomas (20.0%), adenosquamous carcinomas (12.0%), glassy cell carcinoma (4.0%), and leiomyosarcoma (4.0%). The endometrial cancers had the following diagnoses: endometrial carcinoma (79.2%), carcinosarcoma (4.2%), clear cell carcinoma (4.2%), endometrial stromal sarcoma (4.2%), mixed tumor, corpus (4.2%) and serous carcinoma (4.2%). In total, 423 samples from the UCAN cohort were analyzed.

The studies and use of the samples have been approved by the appropriate local ethics committees; Biomovca (Gothenburg University, Ref 139-13), U-CAN (Regionala Etikprövningsnämnden, Uppsala, Dnr: 2016/145), and NSPHS (Regionala Etikprövningsnämnden, Uppsala, Dnr. 2005:325 with approval of an extended project period on 2016-03-19). Written informed consent was obtained from all participating individuals.

### Protein measurements

A custom 11-plex proximity extension assay (PEA)^14^ with a read-out in absolute concentration was used, as in Enroth et al.^13^. Description of the process for combining protein assays into a custom multiplex reaction and the technology used to achieve a readout in absolute concentrations have been described in a white paper^18^. In brief, standard curves with known concentrations are run together with the clinical samples and the standard PEA output is then transformed to absolute concentrations. All the samples were analyzed at the same time and randomized with respect to cohort and diagnosis across plates. All protein measurements were carried out at the Olink Proteomics AB service laboratory in Uppsala, Sweden. Protein concentrations were reported in pg/mL except for KRT19 and MUCIN-16 that were reported in mU/mL. Basic quality control of the data was carried out by Olink Proteomics AB. This procedure flags individual measurements above or below pre-defined limits of quantification. No measurements were reported to be below the limit of detection. A total of 73 measurements, corresponding to 0.6% of the total 12,320 measurements (11*1120 = 12,320), were reported to be as high or above the upper limit of detection and subsequently replaced with the respective upper limit. In the data, 1 of 1120 was replaced for FR-alpha, 7 of 1120 for KRT19, 6 of 1120 for CDH3, 40 of 1120 for MUCIN-16, and 19 of 1120 for SPINT1. Here, 16 (1.4%) of the 1120 samples had one or more of the 11 analytes flagged in the quality control carried out by Olink Proteomics AB and these were removed from further analyses. Seven of these 16 samples (1 malignant, 1 borderline, and 5 benign) were from the Biomovca cohort, and 9 samples (4 malignant, 3 benign, and 2 endometrial cancers) were from the UCAN biobank. No normalization of the protein concentrations was applied.

### Risk score calculation

The raw protein concentrations and individual age were log2-transformed and truncated to the ranges observed in the development of the risk score models as described in Enroth et al.^13^. In total, 296 individual data-points out of 13,440 (11 protein and age in 1120 samples) were truncated for the risk score model. The truncated values were used to calculate the risk scores according to the models previously reported in Supplementary Data 4 of Enroth et al.^13^.

### Statistical analysis

All calculations were done using R^19^ (version 4.0.3). All statistical tests for differences were computed using the Wilcoxon ranked sum test and two-sided unless specified otherwise. Calculation of receiver operating characteristics (ROC) and area under curve (AUC) were performed using the ‘pROC’^20^ R-package. Evaluation of differences in AUC were done using the DeLong’s test as implemented in the ‘pROC’^20^ R-package. Statistical differences in sensitivities and specificities were estimated using a Fisher’s exact test based on counts of false and true positives and negatives. The Cox proportional hazards analyses were conducted using the ‘survival’^21^ R-package (version 3.2-11). The bee-swarm plots were produced using the ‘beeswarm’^22^ R-package (version 0.4.0). All other figures were generated using custom scripts and basic R-functions.

### Reporting summary

Further information on research design is available in the Nature Research Reporting Summary linked to this article.

## Results

### Cohorts and protein biomarker measurements

1120 plasma samples from three separate cohorts were analyzed. The first cohort, Biomovca^15^, was a regional multicenter study (6 hospitals), that included a prospective cohort of patients from both secondary and tertiary referral centers, presenting with an ovarian cyst with and without suspicion of cancer. The second cohort, UCAN^16^, was a clinical prospective cohort consisting of women referred to specialized care with a high suspicion of cancer. The third cohort consisted of age-matched women from a cross-sectional population-based health study, NSPHS (Northern Swedish Population Health study^17^). The sample set from the UCAN biobank also contains serial samples collected from the same woman at time of diagnosis and during treatment and follow-up (Table 1). The samples and cohorts are described in more detail in “Methods” and Table 1. Plasma samples were analyzed using a custom-design, multiplex PEA assay for eleven proteins (MUCIN-16, SPINT1, TACSTD2, CLEC6A, ICOSLG, MSMB, PROK1, CDH3, WFDC2, KRT19, and FR-alpha) (Supplementary Table 1). These proteins were selected based on their inclusion in the risk score models we had previously developed^13^. Absolute concentrations of these proteins were measured with a custom 11-plex PEA (see details in “Methods”). All protein concentrations were reported in pg/mL except for KRT19 and MUCIN-16 which were reported in mU/mL. After stringent quality control (“Methods”), 98.6% (1104) of the samples were included in the further analysis.

### Effect of individual age on protein measurements and risk score

**Fig. 1:**
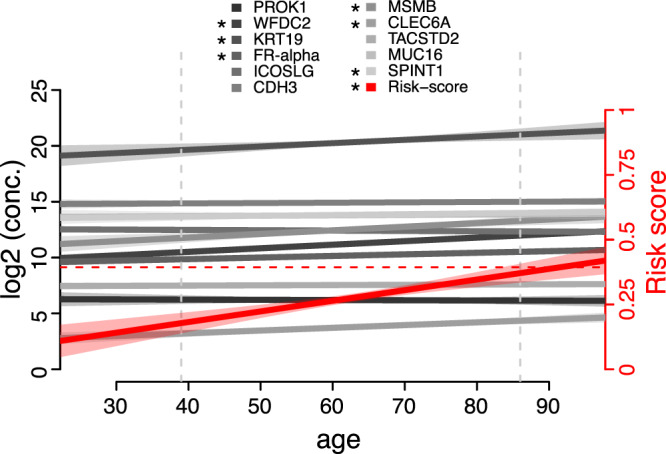
Proteins and risk score in healthy individuals (NSPHS cohort, *N* = 87) in relation to age. Linear models of proteins (left axis) and the risk score (right axis) are indicated in gray scale and red, respectively. The lighter shaded areas represent 95% confidence intervals. Models where age explained a significant (*p* < 0.05) proportion of the observed intra-individual variance in protein concentration or in the risk score are indicated with an * in the legend. The dashed horizontal line corresponds to the “best-point” cut-off for malignancy as described in Enroth et al.^13^. The vertical dashed line corresponds to the lowest and highest individual ages among the samples from the healthy cohort (NSPHS). All protein concentrations are reported in pg/mL except for KRT19 and MUCIN-16 that were reported in mU/mL.

Age is an established factor influencing a large fraction of circulating protein concentrations^23^. The relationship between individual age and levels of the 11 proteins in healthy controls from the NSPHS cohort is shown in Fig. 1. Using 87 control samples we found six of the proteins in our multiplex assay (WFDC2, KRT19, FR-alpha, MSMB, CLEC6A, SPINT1) to be significantly influenced by age using linear regression model, with *p*-values ranging from 7.5 × 10^−3^ to 3.4 × 10^−12^. Although significant, the changes were small in comparison to the observed concentration ranges. For instance, the largest effect was found for MSMB, which increases by 1.02 pg/ml per year on an overall range among healthy women between 1128 and 20,899 pg/ml. The protein concentrations were subsequently log2-transformed and truncated to the observed concentration ranges used in our previously described risk score model (“Methods”)^13^. Based on the healthy age-matched controls from the NSPHS cohort, the risk score models showed significant correlations with individual age (*p*-value = 2.7 × 10^−7^). Age is included in our risk score with a positive coefficient, meaning that age is a positively contributing factor to an increased risk. The per-year increase in the risk score for healthy individuals was however small (4.1 × 10^−3^ per year), and the model remained under the cut-off for malignancy for all point-estimates in the observed age range (Fig. 1). Extrapolation of the linear models indicated that a risk score above the cut-off for malignancy would occur first starting at the age of 91.6 years (Fig. 1).

### The multiplex assay shows high performance at time of diagnosis

To validate our risk score^13^, we studied the performance with respect to separating malignant from benign tumors based on the AUC (Area Under the Curve) and point estimates of sensitivity and specificity for the previously^13^ fixed cut-offs, focusing on (i) sensitivity; (ii) specificity; and (iii) the best-point in the two cohorts Biomovca and UCAN. The ‘best-point’ cut-off was defined as a trade-off between sensitivity and specificity and can be graphically interpreted as the point on the ROC-curve that is closest to perfect classification (e.g., sensitivity and specificity equal to 1.0).

**Fig. 2:**
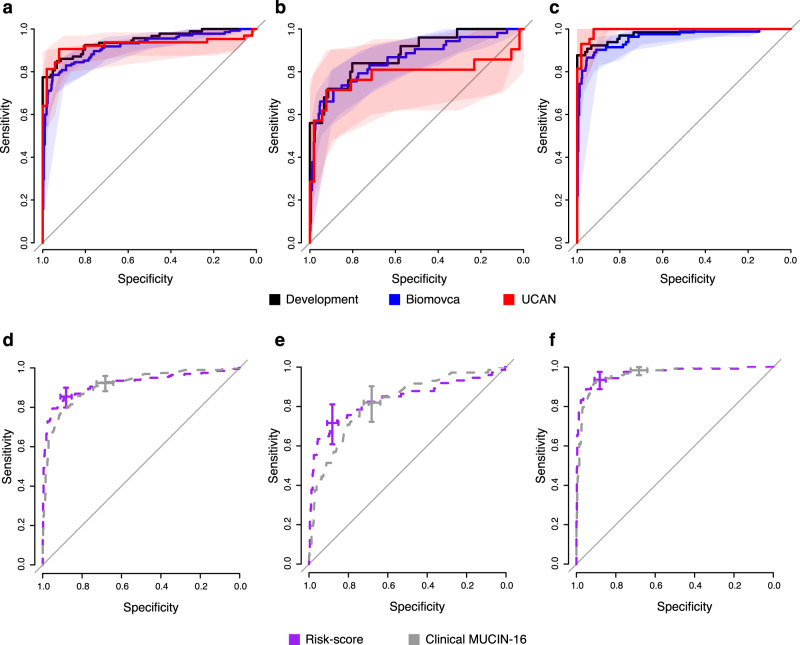
ROC-curves for separating malignant from benign ovarian tumors in the different cohorts. The performance in the development cohort is indicated in black and the Biomovca and UCAN validation cohorts in blue and red, respectively. The solid lines represent the point-estimate and the shaded areas 95% confidence intervals. **a** Separation of malignant tumors stages I–IV (N_UCAN_ = 64, N_Biomovca_ = 135) from benign tumors (N_UCAN_ = 52, N_Biomovca_ = 438). **b** Separation of malignant tumors stages I–II (N_UCAN_ = 21, N_Biomovca_ = 53) from benign tumors (N_UCAN_ = 52, N_Biomovca_ = 438). **c** Separation of malignant tumors stages III–IV (N_UCAN_ = 43, N_Biomovca_ = 82) from benign tumors (N_UCAN_ = 52, N_Biomovca_ = 438). **d** ROC-curves for the risk score (purple) and clinically measured MUCIN-16 (gray), comparing stages I–IV with benign tumors for the combined clinical cohorts (Biomovca and UCAN, *N* = 689). The crosses are centered on the point estimate of the sensitivity and specificity, obtained at the best-point cut-off (risk score, black) and when using 35 U/ml as cut-off for malignancy for MUCIN-16 (blue). The height and width of the crosses illustrate the 95% confidence intervals of sensitivity (y-axis) and specificity (x-axis). **e** and **f** as **d** but for stages I–II (*N* = 564) and III–IV (*N* = 615).

From the Biomovca cohort, we analyzed samples from 610 women, of which 73% had benign tumors, 5% borderline tumors, and 22% were diagnosed with malignant ovarian tumors (Table 1). The ROC-curves for distinguishing between benign tumors and malignant ovarian cancer in the Biomovca and the previous development cohort^13^ are shown in Fig. 2. There was no statistically significant difference in the AUC between the Biomovca and the previous development cohort^13^ for ovarian cancer stages I–IV (DeLong’s test, *p* > 0.24) (Fig. 2a and Table 2). There was also no difference between the Biomovca and the development cohort when comparing early-stage cancers (I and II) and benign tumors (Fig. 2b, DeLong’s test, all *p* > 0.27, Table 2), or late-stage cancers (III and IV) and benign tumors (Fig. 2c, DeLong’s test, all *p* > 0.58, Table 2).

**Table 2:** Comparisons of AUC for separating ovarian malignant and benign tumors between the developmental cohort^13^ and the two validation cohorts for samples collected at the time of diagnosis.

Benign vs stage	Dev.^a^	Biomovca			UCAN			UCAN^b^		
	AUC	AUC	*N*	*p*-val^c^	AUC	*N*	*p*-val^c^	AUC	*N*	*p*-val^c^
I–IV	0.95	0.92	573	0.24	0.93	116	0.56	0.92	80	0.63
I–II	0.89	0.86	491	0.58	0.79	73	0.27	0.77	61	0.45
III–IV	0.98	0.96	520	0.27	0.99	89	0.18	0.99	71	0.33

^a^Developmental cohort from Enroth et al.^13^.

^b^With only the non-overlapping samples with Enroth et al.^13^ (“Methods”).

^c^Two-sided difference compared to the AUC achieved by the models in Enroth et al.^13^.

A second validation was performed using the UCAN cohort (Table 1). The ROC-curves for the UCAN and development cohort^13^ split on cancer stages are shown in Fig. 2. There was no statistical difference in the AUCs for any stage-split compared to our previous development cohort^13^ (Table 2 and Fig. 2). A proportion (*N* = 48, 40%) of the UCAN samples collected at time of diagnosis were used in the second replication cohort in Enroth et al.^13^, and therefore we also analyzed the performance excluding these samples. As for the full dataset, we found no statistical differences in AUC (Table 2). The model had an AUC of 0.920 (Supplementary Table 1) in separating ovarian cancer stages I–IV from benign tumors in this subset of the data. Based on this, we used the full UCAN cohort in the remaining analyses. In addition, we compared the performance of the risk score with the clinically measured MUCIN-16 (CA-125) levels recorded at time of diagnosis (Fig. 2d, e). In this analysis, we combined the benign and malignant samples from the two clinical cohorts (UCAN and Biomovca). There was no difference in the obtained AUCs for any of the stages-splits (Fig. 2d, e, all *q*-values = 1, DeLong’s method, Bonferroni adjusted) between the risk score and using MUCIN-16 alone. When comparing the sensitivity and specificity obtained at the clinically used cut-off at 35 U/ml for MUCIN-16 with the best-point cut-off for the risk score, there was also no statistical difference in the obtained sensitivities (all *q*-values > 0.11, Fishers’s exact test, Bonferroni adjusted, Table 3). There was, however, a significantly higher specificity obtained when using the risk score as compared to MUCIN-16 alone (all *q*-values < 1.3 × 10^−13^, Fishers’s exact test, Bonferroni adjusted, Fig. 2d, e and Table 3).

**Table 3:** Comparisons of AUC, sensitivity, and specificity at time of diagnosis for the risk score and clinical MUCIN-16.

Benign vs stage	AUC			Sensitivity			Specificity		
	Mucin-16	Risk score	*p*-val^a^	Mucin-16	Risk score	*p*-val^b^	Mucin-16	Risk score	*p*-val^b^
I–IV (*N* = 689)	0.89–0.94	0.89–0.95	0.41	0.88–0.96	0.81–0.90	0.037	0.64–0.73	0.85–0.91	4.4 × 10^−14^
I–II (*N* = 564)	0.77–0.88	0.78–0.9	0.40	0.72–0.90	0.61–0.82	0.17	0.64–0.73	0.85–0.91	4.4 × 10^−14^
III–IV (*N* = 615)	0.95–0.98	0.95–0.99	0.73	0.96–1.00	0.89–0.98	0.10	0.64–0.73	0.85–0.91	4.4 × 10^−14^

AUC, sensitivity, and specificity are given as 95% confidence intervals. The comparisons were made using both cohorts (Biomovca and UCAN) with the total number of samples as indicated in the first column.

^a^DeLong’s test.

^b^Fishers’ exact test.

**Fig. 3:**
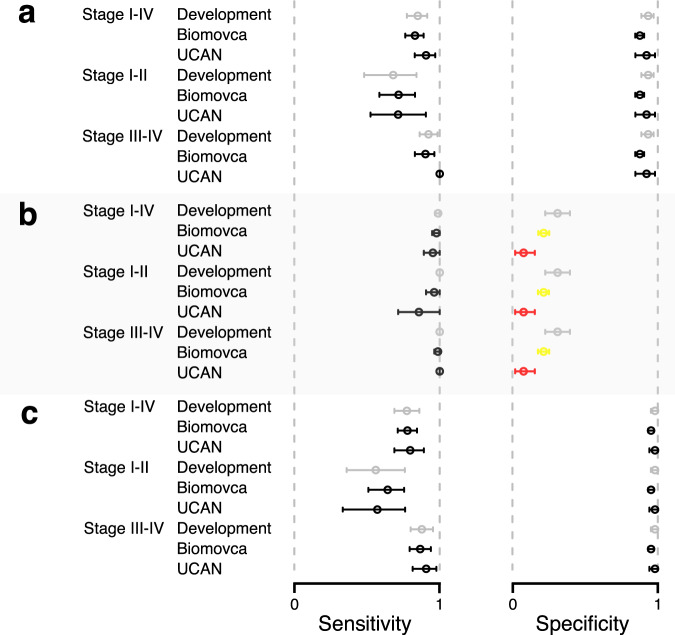
Comparisons of sensitivity and specificity in the cohorts at the three cut-offs developed earlier^13^. **a** Best-point, **b** focus-on-sensitivity, and **c** focus-on-specificity. The circles represent point-estimate and horizontal line the 95% confidence interval. The light gray colored marks represent the development cohort from Enroth et al.^13^ and the black, yellow, or red colored marks represent results in the two validation cohorts. Yellow marks are nominally significant (*p* < 0.05, two-sided Fishers’ exact test) differences compared to the development results, while red marks represent significant differences also after adjustment for multiple hypothesis testing (Bonferroni, *p* < 0.05/36). The Biomovca analysis was based on comparisons of 438 benign, 53 stage I–II, and 82 stage III–IV samples. The UCAN analysis was based on comparisons of 52 benign, 21 stage I–II, and 43 stage III–IV samples.

We next evaluated the performance of the three fixed cut-offs compared with the results obtained in the development cohort^13^. The point-estimate and 95% CI for the Biomovca and development cohort for each type of cut-off is shown in Fig. 3. The best-point cut-off in Biomovca was not statistically different from estimates of sensitivity (all *p*-values > 0.72) or specificity (all *p*-values > 0.12, Fig. 3a and Supplementary Table 1) for the development cohort. There was also no statistical difference between the Biomovca cohort and the development cohort with respect to the sensitivity of the models (all *p*-values > 0.65, Supplementary Table 1) for the focus-on-sensitivity (Fig. 3b) and no difference in specificity for the focus-on-specificity (Fig. 3c) (all *p*-values > 0.28, Supplementary Table 1). The model did show a lower nominally significant (*p* < 0.041) specificity for the focus-on-sensitivity cut-off (Fig. 3b and Supplementary Table 1). The sensitivity for the focus-on-specificity model did not differ significantly between the Biomovca and the development cohort (Fig. 3c, all *p*-values > 0.62, Supplementary Table 1). All point estimates, confidence intervals and *p*-values comparing the performance in the developmental and Biomovca cohorts are presented in Supplementary Table 1. Among the borderline samples in the Biomovca cohort, 17, 33, and 87% of the samples had a risk score indicating malignancy at the focus-on-specificity, best-point, and focus-on-sensitivity cut-offs, respectively. We also compared the UCAN cohort and the development cohort with respect to the obtained sensitivity and specificity at the three fixed cut-offs. As above, there was no statistical difference in any of the analyses in relation to sensitivity (all *p*-values > 0.088, Fig. 3a–c and Supplementary Table 1). The model did show a nominally significantly (*p* < 1.1 × 10^−3^) lower specificity for the focus-on-sensitivity cut-off (Fig. 3b and Supplementary Table 1). This difference remained significant after adjustment for multiple hypothesis testing (*q* < 0.041, Bonferroni). All other specificities were not significantly different (Fig. 3a, c, all *p*-values > 0.75, Supplementary Table 1). All point estimates, confidence intervals and *p*-values comparing the performance in the developmental and validation cohorts are presented in Supplementary Table 1. In summary, the risk score showed similar performance in the two independent cohorts as in the original development cohort.

### Risk scores in healthy individuals and in other gynecological cancers

**Fig. 4:**
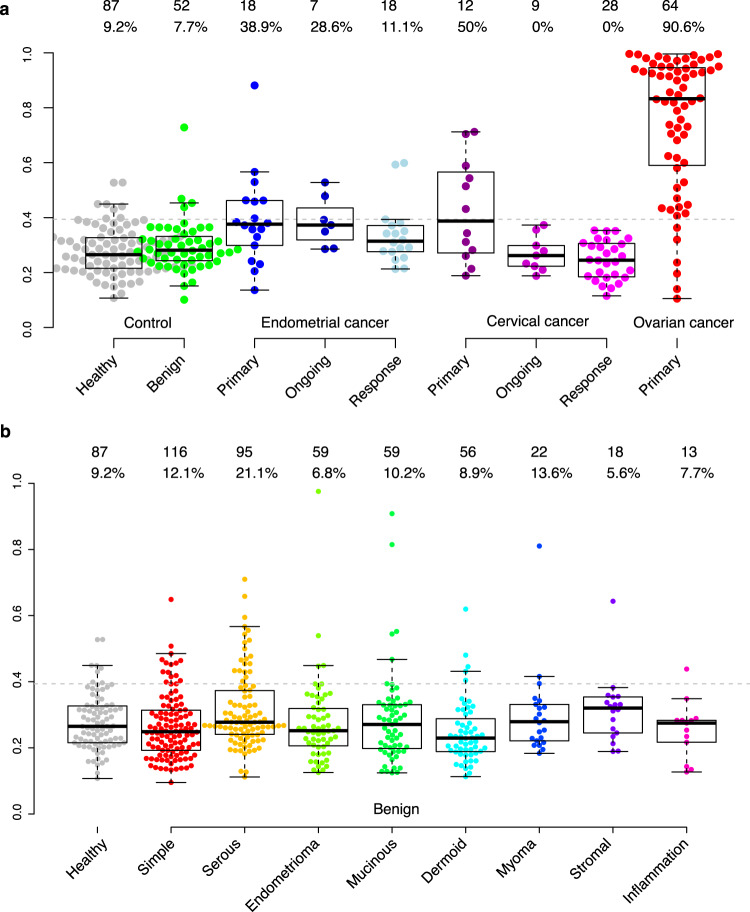
Risk score levels across multiple cancers, benign histology, and controls. **a** Risk score for healthy women and those with benign tumors, or endometrial, cervical and ovarian cancer (stages I–IV). The dotted line indicates the cut-off for malignancy at the ‘best-point’ established for ovarian cancer. The numbers above each group represent the number of samples and the percent of samples that are above the cut-off. All samples except the healthy (NSPHS) are from the UCAN-cohort. **b** Risk scores in healthy individuals and different benign histologies. The dotted line indicates the cut-off for malignancy at the ‘best-point’ established for ovarian cancer. The numbers above each group represent the number of samples and the percent of samples that are above the cut-off. All samples except the healthy (NSPHS) are from the Biomovca cohort. **a**, **b** The top and the bottom of the box represent the 25th and 75th percentile and the band inside the box the median value. The whiskers are calculated as 1.5x the interquartile range.

Our risk score model was developed using malignant and benign tumor samples^13^, and to broaden the spectra, we therefore examined the risk score also in symptom free, healthy women and women diagnosed with endometrial or invasive cervical cancer (Table 1 and Fig. 4a). Healthy women had on average lower risk scores than those with benign tumors, although the difference was not statistically significant (*p* < 0.28, two-sided Wilcoxon ranked test). There was also no significant difference between healthy women and those with benign tumors in the percentage of samples above the cut-off for malignancy (*p* = 1, Fisher’s exact test, 9.2% and 7.7%, respectively). As a comparison, the fraction of samples above the malignancy cut-off for ovarian cancer stages I–IV was 90.6% (Fig. 4a). Thus, the risk score for women with benign tumors was similar to that of healthy women.

For cervical and endometrial cancers, the risk score was calculated at time of diagnosis, and during and after completion of treatment. For cervical cancer 50.0% of samples were above the ovarian cancer malignancy cut-off at the time of diagnosis, and this was reduced to 0% during or after treatment (Fig. 4a). For endometrial cancer, 38.9% of the samples collected at time of diagnosis were above the cut-off for ovarian cancer malignancy, and this was reduced to 28.6% during treatment and 11.0% after treatment. (Fig. 4a). Thus, the risk score detects a fraction of cervical cancer and endometrial cancer at diagnosis, while at follow-up, the cancer specificity of the risk score is increased.

Using the benign samples from the Biomovca cohort (Table 1), we further compared the risk scores stratified on the different available histologies. When compared to healthy women, there were no statistical difference in distribution of risk scores in the benign histologies, except for dermoid cysts which had lower scores (*p* = 8.5 × 10^−3^, Wilcoxon’s ranked test). This difference was not significant after adjustment for multiple hypothesis testing (Bonferroni, *q* = 6.8 × 10^−2^). Next, we calculated the fraction of observations in each group that was above the cut-off and found between 5.6 and 21.1% (Fig. 4b). Compared with the 9.2% found among healthy women, one group, serous cysts, had a nominally significantly higher proportion (*p* = 3.9 × 10^−2^, Fishers’s exact test), but this difference did not remain significant after adjustment for multiple hypothesis testing (Bonferroni, *q* = 0.69).

### Risk score at follow-up is informative of treatment outcome

**Fig. 5:**
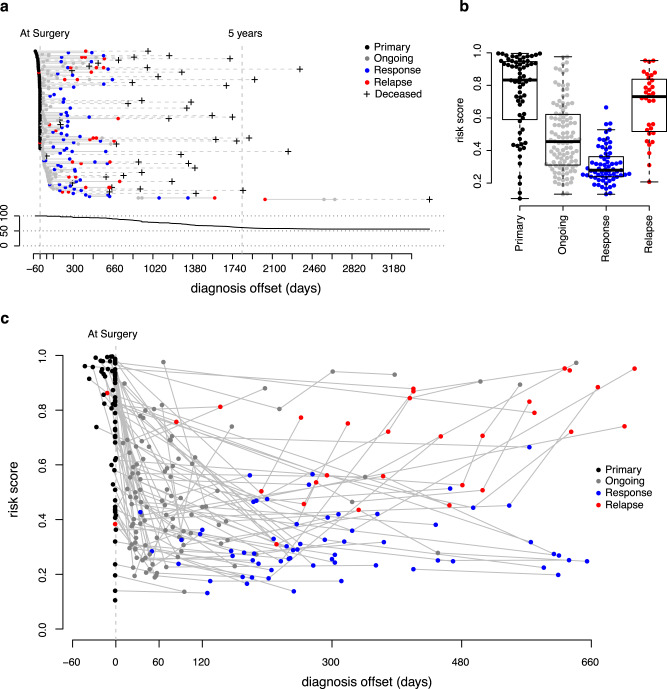
Overview of collection of samples in relation to time of surgery in the UCAN cohort. **a** The upper panel shows the sample series from each patient on separate horizontal lines, with the color of the dots referring to where in the treatment progression the samples was collected: Primary sample (*N* = 64, black), Treatment ongoing (*N* = 108, gray), Response to treatment (*N* = 65, blue), and Relapse/progression (*N* = 32, red) (see “Methods”). A black cross indicates the timepoint when a patient died. The lower panel indicates the overall survival (in %) in the cohort. **b** Distribution of risk scores at the four clinical stages above. The top and the bottom of the box represent the 25th and 75th percentile and the band inside the box the median value. The whiskers are calculated as 1.5x the interquartile range. Outliers were omitted from the boxplots. **c** Risk score development (y-axis) for individual patients over a 2-year period (x-axis), starting from time of diagnosis. Sampling events are colored to represent the four categories above. Gray lines connect sampling events for the same patient.

Our ovarian cancer risk score was further calculated based on serial samples from the UCAN cohort. This included samples collected when the tumor was diagnosed but prior to commencing treatment (“Primary baseline sample”); when commencing surgery or chemotherapy until the end of the course of treatment (“Treatment ongoing”); during follow-up after completed treatment with a group of women in partial or complete remission (“Response to treatment”); and, finally, when cancer had recurred after an initial positive response to treatment or when cancer was progressing (“Relapse/progression”). Figure 5a shows the samples collected from each woman relative to time of surgery, and Fig. 5b the risk scores in the four groups. The risk score was dramatically reduced upon treatment, in particular for women responding to treatment and rose again in women with relapse/progression (Fig. 5b). All pairwise comparisons of groups showed nominally significant differences in risk score (*p* < 3.9 × 10^−2^, Supplementary Table 2) and all, except for the comparison between the samples taken at diagnosis and at relapse, were also significant after correction for multiple hypothesis testing (Bonferroni, *q* < 1.1 × 10^−4^, Supplementary Table 2). These results suggest strongly that the risk score is reflecting the course of the disease.

Next, we analyzed changes in risk score during a 2-year period for individual women with multiple samples in the UCAN cohort (Fig. 5c). In order to identify pattern templates to which a patient’s individual observations could be assigned we used series with four or more samples per patient collected within a 2-year period from diagnosis. Fifteen patients with at least four samples were identified and the risk scores of these sample series were modeled across the sampling range (time) using a cubic spline. These fifteen models were then re-sampled at fixed time intervals in order to create a dataset with common time-points following diagnosis. The risk score at these time-points was then used as input to kmeans-clustering with four centers. All individual sample series, regardless of number of samples, were then assigned to either of the clusters using a Euclidean distance metric.

**Fig. 6:**
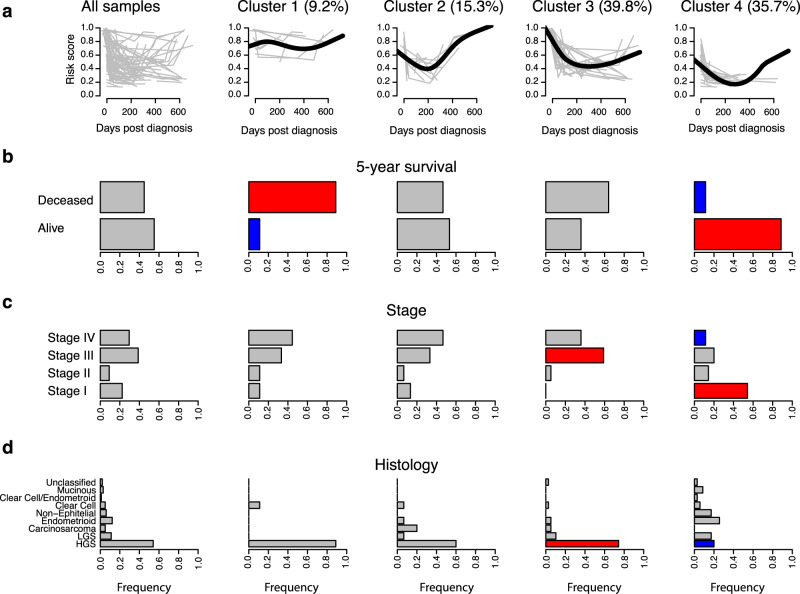
Time-series clusters based on the risk score in the UCAN cohort. The risk score trajectories of the clusters correlate with clinical history and treatment outcome of patients. **a** From left to right, individual risk scores across sample occasions are illustrated in all samples (leftmost panel) and then for the four individual clusters. Cluster-centers are illustrated in solid black and individual profiles assigned to that cluster in gray. A total of 98 samples series were analyzed. Cluster 1 had 9 patients, cluster 2 had 15 patients, cluster 3 had 39 patients, and cluster 4 had 35 patients. Proportion (%) of all patients assigned to each cluster is indicated above each panel. **b** Proportion of alive and deceased women during the 2-year follow-up period among all samples (leftmost panel) and individual clusters. **c** Distribution of ovarian cancer stages (I–IV) at time of diagnosis in all samples (leftmost panel) and each cluster. **d** Distribution of tumor histology classifications (“Methods”) at time of diagnosis in all patients (leftmost panel) and in each cluster. **b**–**d** Red and blue bars indicate a nominally significant (*p* < 0.05, two-sided Fisher’s exact test) higher or lower frequency, respectively, as compared to the total distribution (leftmost panel).

The four time-series clusters based on these templates are shown in Fig. 6a. This process assigned between 9.2 and 39.8% of individual women’s sample series to one of the four clusters (Fig. 6a). The risk score trajectory of the clusters indicates four different clinical response patterns. About 10% of the women (Cluster 1, panel 2 from the left) showed a consistent high-risk score from date of diagnosis and no change during treatment, indicating lack of treatment response. For another 15% of the women (Cluster 2) the risk score was reduced during treatment, and then rapidly returned to its initial high value after completion of treatment, indicating rapid relapse. The largest group (Cluster 3), including almost 40% of the women, showed a rapid decrease from a high-risk score during or after treatment to a lower score, followed by a slow but steady increase post-treatment. Finally, Cluster 4, with 36% of the women, had a moderately high-risk score at diagnosis which dropped during treatment, but then increased slowly during follow-up. Clusters 1, 2, and 3 all had a higher proportion of women who died during the follow-up period (Fig. 6b), a higher proportion of ovarian cancer stages III and IV (Fig. 6c), and a higher proportion of HGS histology (Fig. 6d), when compared to cluster 4 or the overall mean. Thus, the risk score trajectories of the clusters correlate with the clinical history and treatment outcome of patients.

### Risk score at time of diagnosis is predictive of 5-year survival

Using the risk score at time of diagnosis, we modeled the relative contribution of the risk score, individual age, BMI, clinically measured MUCIN-16 (CA-125) and cancer stage at time of diagnosis on overall 5-year survival, using a Cox proportional hazards regression model, based on the UCAN cohort. Stage was found to be the strongest predictive variable (*p* < 3.5 × 10^−4^, Bonferroni adjusted *q*-value 1.7 × 10^−3^) with our risk score being the second strongest, reaching nominal significance (*p* < 1.8 × 10^−2^, *q*-value > 0.05). Neither age, BMI nor clinical CA-125 at time of diagnosis had any statistically significant effect (all *p* > 0.77, Supplementary Table 3). In a second model with only risk score, age, BMI and clinical CA-125, risk score was the only significant variable (*p* < 2.0 × 10^−3^, Bonferroni adjusted *q*-value 7.9 × 10^−3^, all other *p*-values > 0.77, Supplementary Table 3). In both models, a higher cancer stage or risk score predicted a lower 5-year survival. Lastly, we built a model with risk score, age, BMI and clinical CA-125 using only patients with late-stage cancer (stages III or IV). For models using only stage III, the risk score was nominally significant (*p* = 0.045, all other *p*-values > 0.21, Supplementary Table 3), while for stage IV, lower BMI was found to reflect lower survival (*p* < 4.8 × 10^−3^, Bonferroni adjusted *q*-value 1.9 × 10^−2^) with no other variables reaching statistical significance (*p* > 0.26, Supplementary Table 3). Thus, the 5-year survival analysis indicates that, after cancer stage, the risk score, but not CA-125 alone, was the second most predictive variable of survival.

## Discussion

In the diagnosis of women with adnexal tumors, diagnostic surgery with curative intention is used to confirm indications by TVU. In Sweden, about 75% of the women evaluated by diagnostic surgery have benign ovarian tumors (benign cysts)^15^ and diagnostic surgery is associated with potential surgical complications, side-effects on fertility, and induced menopause^24^. We have developed a risk score model based on a multiplex plasma protein assay^13^ that can be used to distinguish between benign tumors and ovarian cancer in women with adnexal ovarian masses, and thereby reduce the need for diagnostic surgery. The performance of the risk score model was validated in two independent clinical cohorts from different parts of Sweden. The model was used with fixed parameters and fixed cut-offs and showed essentially the same performance with respect to sensitivity and specificity in the two new cohorts as in cohorts previously used to develop the model. The protein measurements from the validation cohorts were fed directly into the models without any prior normalization or transformation of the data. The samples used have been collected at several different healthcare centers and hospitals across Sweden, using the local collection protocols without any prior synchronization of collection protocols between the three cohorts used. The Biomovca cohort include a series of secondary care centers in the region of Western Sweden^15^ from which samples were transported to the central biobank. The samples selected were not limited to a particular subset of histology categories but were instead reflective of the distribution in Sweden today. It should be noted that a proportion (40%) of the samples collected at time of diagnosis from the UCAN cohort used here were included in the second replication cohort in our previous study^13^. When analyzed separately, these samples (40%) did not show any increase in performance compared to the non-overlapping proportion (60%) and we mainly reported replication results from the combine set. In our previous study, these samples were not at any point used to select the proteins in the combine biomarker panel nor to train the coefficients of the model used here for calculation of the risk score. The overlapping proportion was also previously analyzed with a standard PEA reporting in the relative NPX-scale and not in absolute concentrations as used here. Apart from clinical conditions, from a technical standpoint, pre-clinical conditions, such as storage time can affect the level of some plasma proteins^25^. For instance, the measured levels of MUCIN-16 in frozen (−70 °C) plasma have been shown to increase with prolonged storage time across several decades^25^. Since the clinical samples in the current study were collected over a shorter time-period (2012−2018) and the healthy cohort in 2006, and, still more important, the impact of storage time on the remaining proteins in the risk score is unknown, we elected not to apply adjustments related to storage time. The validation shows that the assay is robust and delivers similar performance across cohorts and pre-analytic handling, including samples collected at different times and in different clinical settings. Over 90% of the women in the validation cohorts with benign tumors had risk scores below the cut-off for malignancy, and therefore would not have needed immediate diagnostic surgery. In comparison with clinically measured MUCIN-16 (CA-125), our risk score showed a higher specificity at a retained sensitivity for classification of benign tumors at time of diagnosis.

We also examined the risk score at different stages of clinical management and cancer development. Serial samples were available from before treatment initiation, during ongoing treatment, when treatment was completed, and during relapse and/or cancer progression. Once treatment was initiated, the risk score dropped and continued to do so in patients responding to treatment. After completion of treatment, the risk score in most women increased, but at different rates and with different final levels. In general, women in relapse or whose disease was getting worse had high-risk scores. Taken together the risk score followed the clinical course of the disease and the treatment outcome.

In order to be able to follow the risk score and disease development in individual women, we studied patients with multiple sampling occasions at time of diagnosis, treatment, and follow-up to derive common risk score trajectories. These trajectories where then used as templates to which the data of other patients in the study could be matched. We identified four main risk score trajectories running from date of diagnosis to the 2-year follow-up. The four trajectories (clusters) correspond to common clinical responses such as: Cluster 1 (patients with no treatment response); Cluster 2 (patients showing an initial reduction of the risk score, indicative of a positive treatment response, followed by rapid relapse); Cluster 3 (patients with a rapid decrease of the risk score during or after treatment, and an increase post-treatment), and, finally, Cluster 4 (patients with good treatment response but nevertheless a slowly increasing risk score during follow-up). The identification of trajectories is obviously a simplification of individual clinical histories and more than four clusters could be defined, but the analysis indicates that the risk score does follow, and capture, major clinical scenarios. The four clusters differ with respect to tumor stage, histology, and survival during follow-up, consistent with the most important clinical scenarios. In support of this, the risk score at diagnosis is, after tumor stage, the second strongest predictor of 5-year survival. In the same analysis, clinically measured MUCIN-16 at time of diagnosis did not show any statistically significant predictivity.

In this study, we focused on the ability of the assay to reduce the need for diagnostic surgery when managing women with adnexal ovarian mass, and on the possibility of predicting the clinical outcome of treatment. However, the only way to improve the prognosis of ovarian cancer would be to introduce means for detection of early-stage cancer through screening. In the present study we were unable to evaluate the performance of the assay before diagnosis, and therefore its potential for population screening. Nevertheless, the dramatic reduction of the risk score upon initiation of treatment and its increase during relapse demonstrates that the assay is able to reflect tumor burden, and has the potential to be informative also before diagnosis. Further studies are needed to examine this latter possibility. In general, screening could be in the form of yearly or biyearly testing in the high-risk group of women at relevant ages. If this is carried out using home-sampling devices, burdening of primary healthcare centers with routine sample collections could be avoided. Such longitudinal sample collection among higher-risk groups would also enable the use of individual thresholds for identification of early-stage ovarian cancer. These are likely to be more sensitive than general population-based thresholds^26^. In addition, an optimal screening program should identify women before the cancer has developed or before the transformation from identified precursor lesions to cancer is complete. Investigations into this would ideally require both tissue samples from the developing tumor, including precursor states, for accurate staging and relevant peripheral tissue for screening purposes. Although there are ongoing collections of peripheral tissue (blood) such as the United Kingdom Collaborative Trial of Ovarian Cancer Screening (UKCTOCS)^27^ for screening purposes, obtaining the precise tumor development stage at the time of collection in women with no presenting symptoms is not possible. A more realistic goal, which should improve survival rates, would be to target investigations into early discovery of the cancer. It is, however, important to bear in mind that ovarian cancers presenting in early stage often have a less aggressive histology (type 1 tumors) and clinical appearance than those diagnosed in later stage (type 2 tumors), and a direct comparison between groups should be made with caution^28^. Here, and in our previous study^13^, the aim was to search for a clinically usable protein biomarker signature that would transcend these differences and signal the cancer regardless of the origin and stage. Future studies seeking biomarkers for early detection of ovarian cancer could target the different types separately and compare them both to healthy controls and to patients diagnosed with benign tumors. This was recently illustrated in an exploratory study based on a broad characterization of plasma proteins specifically identifying different combinations of biomarkers for late and early-stage cancer compared to benign tumors^29^.

Here, we also compared women with benign tumors (cysts) with healthy age-matched women and found the risk score to be similar in these two groups, albeit slightly higher in women with benign tumors. This is in accordance with the assay providing a sensitive evaluation of ongoing processes that may result in cancer. The risk score was also shown to be elevated at diagnosis in women with endometrial or cervical cancer. Cervical cancer is normally handled separately through screening and/or a molecular test for the presence of high-risk human papilloma virus (HPV). For endometrial cancer, TVU in women with symptoms is often sufficiently indicative, and no screening is presently in place. However, if our assay were to be employed in population screening, the cancer specificity would have to be more carefully considered.

The performance of our assay was robust across clinical cohorts, but for clinical use even a small increase in sensitivity and specificity would be beneficial. The ROMA-index, as originally suggested, achieved a sensitivity of 0.92 at a specificity of 0.75 in post-menopausal women and a sensitivity of 0.77 at a specificity of 0.75 in pre-menopausal women^30^. A recent meta-analysis of performance of the ROMA-index in both pre- and post-menopausal women suggests an overall sensitivity in the range of 0.88 to 0.93, and a specificity in the range of 0.89 to 0.94^31^. That study^31^ showed variation in the performance between cohorts and^26^ could not rule out biases in the reported results, either due to the underlying distribution of biological samples in the participating studies or in the meta-analysis performed. Apart from MUCIN-16 and WFDC2 that are used in the ROMA-index, other studies have indicated that additional protein biomarkers can be informative for e.g., early diagnosis or screening. Russel and colleagues^26^ combined MUCIN-16, Vitamin K-dependent protein Z (PROZ), phosphatidylcholine-sterol acyltransferase (LCAT) and C-reactive protein (CRP) into a multiplex biomarker panel that, when used against a patient’s own baseline, displayed promise in detecting ovarian cancer 1–2 years earlier than current diagnostic methods. Our risk score was developed across both pre- and post-menopausal women based on measuring 593 proteins in plasma^13^, and using a model which selected proteins based on how well they discriminated between cases and control in combination, regardless of their univariate performance. Indeed, some of the eleven proteins did not show any statistical evidence of separating cases and controls on their own^13^. Recent technological advancements in proteomics allow for highly specific analyses of thousands of proteins from each sample^32^. Further studies of additional proteins could increase the performance of the risk score model in relation to ovarian cancer, as well as result in the identification of additional biomarkers with high specificity for the individual gynecological cancers. This is a reasonable assumption based on a previous study we conducted^33^ where we studied plasma biomarkers in both ovarian and endometrial cancer compared to benign tumors. In that study, 16 proteins were detected for endometrial cancer and 15 for ovarian cancer, but only 4 were selected for both.

In conclusion, we have shown that the performance of the risk score model is robust across cohorts. Also, the risk score pattern after diagnosis follows the common clinical responses during treatment and relapse/progression and may be useful in monitoring clinical developments during follow-up.

## Supplementary information

Supplementary Material

Supplementary Data 1

Reporting Summary

Description of Additional Supplementary Files

## Data Availability

The datasets generated during the current study are available from the authors on reasonable request. Source data for the figures are available as Supplementary Data 1.
