# Supplementary material for: Data-driven analysis of a validated risk score for ovarian cancer identifies clinically distinct patterns during follow-up and treatment
Source: Commun Med (Lond). 2022 Oct 1;2:124. doi: 10.1038/s43856-022-00193-6 (PMC9526736; doi:10.1038/s43856-022-00193-6)
Supplement: Supplementary file 1 — Supplementary Material [file 43856_2022_193_MOESM1_ESM.pdf]

Supplementary Table 1. Performance comparison with the previous cohort.

Column names:
"val" denotes values from the validation cohorts, and "dev" in the previous development cohort (Enroth et al, Communications Biology, 2019).
"SE" and "SP" denotes "Sensitivty" and "Specificity" respectively.
"pe" is "point estimate" and "l"/"h" is the "low" and "high" ranges of 95% confidence intervals.
"ucan" and "biomovca" are the two validation cohorts, "ucan_new" represent the analysis restricted to only non-overlapping sampels with Enroth et al 2019, Communications Biology.

**cohort**	**model**	**focus**	**stages**	**devAUCpe**	**devAUCl**	**devAUCh**	**valAUCpe**	**valAUCl**	**valAUCh**	**dAUCpval**	**devSEl**	**devSEpe**	**devSEh**	**devSPl**	**devSPpe**	**devSPh**	**valSEl**	**valSEpe**	**valSEh**	**valSPl**	**valSPpe**	**valSPh**	**dSEpvalFisher**	**dSPpvalFisher**
ucan	11p+a	bp	12	0,885	0,806	0,964	0,787	0,63	0,943	0,273642343	0,48	0,68	0,84	0,887	0,934	0,972	0,524	0,714	0,905	0,846	0,923	0,981	1	0,75204951
ucan	11p+a	fse	12	0,885	0,806	0,964	0,787	0,63	0,943	0,273642343	1	1	1	0,226	0,311	0,396	0,714	0,857	1	0,019	0,077	0,154	0,087615283	0,001134339
ucan	11p+a	fsp	12	0,885	0,806	0,964	0,787	0,63	0,943	0,273642343	0,36	0,56	0,76	0,953	0,981	1	0,333	0,571	0,762	0,942	0,981	1	1	1
biomovca	11p+a	bp	12	0,885	0,806	0,964	0,856	0,791	0,921	0,57880955	0,48	0,68	0,84	0,887	0,934	0,972	0,585	0,717	0,83	0,845	0,877	0,906	0,793173391	0,121319396
biomovca	11p+a	fse	12	0,885	0,806	0,964	0,856	0,791	0,921	0,57880955	1	1	1	0,226	0,311	0,396	0,906	0,962	1	0,176	0,215	0,251	1	0,040601093
biomovca	11p+a	fsp	12	0,885	0,806	0,964	0,856	0,791	0,921	0,57880955	0,36	0,56	0,76	0,953	0,981	1	0,509	0,642	0,755	0,934	0,954	0,973	0,618767284	0,278092714
both	11p+a	bp	12	0,885	0,806	0,964	0,838	0,776	0,9	0,358918244	0,48	0,68	0,88	0,887	0,934	0,981	0,608	0,716	0,811	0,853	0,882	0,91	0,800955675	0,125392143
both	11p+a	fse	12	0,885	0,806	0,964	0,838	0,776	0,9	0,358918244	1	1	1	0,226	0,311	0,406	0,865	0,932	0,986	0,167	0,2	0,237	0,325912602	0,014217899
both	11p+a	fsp	12	0,885	0,806	0,964	0,838	0,776	0,9	0,358918244	0,36	0,56	0,76	0,953	0,981	1	0,514	0,622	0,73	0,939	0,957	0,976	0,639769238	0,401713337
ucan	11p+a	bp	34	0,977	0,956	0,998	0,993	0,983	1	0,184328873	0,862	0,923	0,985	0,887	0,934	0,972	1	1	1	0,846	0,923	0,981	0,154698694	0,75204951
ucan	11p+a	fse	34	0,977	0,956	0,998	0,993	0,983	1	0,184328873	1	1	1	0,226	0,311	0,406	1	1	1	0,019	0,077	0,154	1	0,001134339
ucan	11p+a	fsp	34	0,977	0,956	0,998	0,993	0,983	1	0,184328873	0,8	0,877	0,954	0,953	0,981	1	0,814	0,907	0,977	0,942	0,981	1	0,759684248	1
biomovca	11p+a	bp	34	0,977	0,956	0,998	0,958	0,931	0,984	0,265480085	0,862	0,923	0,985	0,887	0,934	0,972	0,829	0,902	0,963	0,845	0,877	0,906	0,77437903	0,121319396
biomovca	11p+a	fse	34	0,977	0,956	0,998	0,958	0,931	0,984	0,265480085	1	1	1	0,226	0,311	0,396	0,963	0,988	1	0,176	0,215	0,253	1	0,040601093
biomovca	11p+a	fsp	34	0,977	0,956	0,998	0,958	0,931	0,984	0,265480085	0,8	0,877	0,954	0,953	0,981	1	0,793	0,866	0,939	0,934	0,954	0,973	1	0,278092714
both	11p+a	bp	34	0,977	0,956	0,998	0,969	0,95	0,987	0,549871768	0,862	0,923	0,985	0,887	0,934	0,981	0,888	0,936	0,976	0,851	0,882	0,91	0,766737057	0,125392143
both	11p+a	fse	34	0,977	0,956	0,998	0,969	0,95	0,987	0,549871768	1	1	1	0,226	0,311	0,406	0,976	0,992	1	0,163	0,2	0,237	1	0,014217899
both	11p+a	fsp	34	0,977	0,956	0,998	0,969	0,95	0,987	0,549871768	0,785	0,877	0,954	0,953	0,981	1	0,816	0,88	0,936	0,939	0,957	0,973	1	0,401713337
ucan	11p+a	bp	1234	0,945	0,913	0,976	0,925	0,868	0,982	0,562065317	0,774	0,849	0,914	0,877	0,934	0,972	0,828	0,906	0,969	0,846	0,923	0,981	0,338814355	0,75204951
ucan	11p+a	fse	1234	0,945	0,913	0,976	0,925	0,868	0,982	0,562065317	0,968	0,989	1	0,226	0,311	0,406	0,891	0,953	1	0,019	0,077	0,154	0,305010913	0,001134339
ucan	11p+a	fsp	1234	0,945	0,913	0,976	0,925	0,868	0,982	0,562065317	0,688	0,774	0,86	0,953	0,981	1	0,688	0,797	0,891	0,942	0,981	1	0,844380151	1
biomovca	11p+a	bp	1234	0,945	0,913	0,976	0,918	0,886	0,95	0,243972824	0,774	0,849	0,914	0,887	0,934	0,972	0,763	0,83	0,889	0,845	0,877	0,906	0,719015877	0,121319396
biomovca	11p+a	fse	1234	0,945	0,913	0,976	0,918	0,886	0,95	0,243972824	0,968	0,989	1	0,226	0,311	0,396	0,948	0,978	1	0,178	0,215	0,253	0,647134717	0,040601093
biomovca	11p+a	fsp	1234	0,945	0,913	0,976	0,918	0,886	0,95	0,243972824	0,688	0,774	0,86	0,953	0,981	1	0,711	0,778	0,844	0,934	0,954	0,973	1	0,278092714
both	11p+a	bp	1234	0,945	0,913	0,976	0,92	0,892	0,948	0,253507594	0,774	0,849	0,914	0,887	0,934	0,972	0,804	0,854	0,899	0,851	0,882	0,91	1	0,125392143
both	11p+a	fse	1234	0,945	0,913	0,976	0,92	0,892	0,948	0,253507594	0,968	0,989	1	0,226	0,311	0,396	0,945	0,97	0,99	0,167	0,2	0,237	0,437100814	0,014217899
both	11p+a	fsp	1234	0,945	0,913	0,976	0,92	0,892	0,948	0,253507594	0,688	0,774	0,849	0,953	0,981	1	0,724	0,784	0,839	0,937	0,957	0,976	0,879847414	0,401713337
ucan_new	11p+a	bp	12	0,885	0,806	0,964	0,774	0,5	1	0,445421736	0,48	0,68	0,84	0,887	0,934	0,981	0,444	0,778	1	0,846	0,923	0,981	0,692066097	0,75204951
ucan_new	11p+a	fse	12	0,885	0,806	0,964	0,774	0,5	1	0,445421736	1	1	1	0,226	0,311	0,406	0,444	0,778	1	0,019	0,077	0,154	0,064171123	0,001134339
ucan_new	11p+a	fsp	12	0,885	0,806	0,964	0,774	0,5	1	0,445421736	0,36	0,56	0,76	0,953	0,981	1	0,333	0,667	0,889	0,942	0,981	1	0,704115684	1
ucan_new	11p+a	bp	34	0,977	0,956	0,998	0,99	0,975	1	0,332096663	0,862	0,923	0,985	0,887	0,934	0,981	1	1	1	0,846	0,923	0,981	0,583319729	0,75204951
ucan_new	11p+a	fse	34	0,977	0,956	0,998	0,99	0,975	1	0,332096663	1	1	1	0,226	0,311	0,406	1	1	1	0,019	0,077	0,154	1	0,001134339
ucan_new	11p+a	fsp	34	0,977	0,956	0,998	0,99	0,975	1	0,332096663	0,8	0,877	0,954	0,953	0,981	1	0,684	0,842	1	0,942	0,981	1	0,705703512	1
ucan_new	11p+a	bp	1234	0,945	0,913	0,976	0,92	0,827	1	0,632703996	0,774	0,849	0,914	0,887	0,934	0,972	0,821	0,929	1	0,846	0,923	0,981	0,356410798	0,75204951
ucan_new	11p+a	fse	1234	0,945	0,913	0,976	0,92	0,827	1	0,632703996	0,968	0,989	1	0,226	0,311	0,396	0,821	0,929	1	0,019	0,077	0,154	0,133446767	0,001134339
ucan_new	11p+a	fsp	1234	0,945	0,913	0,976	0,92	0,827	1	0,632703996	0,688	0,774	0,86	0,953	0,981	1	0,643	0,786	0,929	0,942	0,981	1	1	1

Supplementary Table 2. Comparison of risk-score in the broad groups.

**Group 1**	**Group 2**	**model**	**mean Group 1**	**mean Group 2**	**pvalue (two-sided Wilcoxon ranked test)**	**pvalue (Bonferroni adjusted)**
primary	respons	11p+a	0,74844717	0,310194232	4,90E-16	2,94E-15
respons	relapse	11p+a	0,310194232	0,678670254	6,73E-12	4,04E-11
primary	ongoing	11p+a	0,74844717	0,484698382	1,23E-10	7,38E-10
respons	ongoing	11p+a	0,310194232	0,484698382	1,02E-08	6,10E-08
ongoing	relapse	11p+a	0,484698382	0,678670254	1,91E-05	0,000114615
primary	relapse	11p+a	0,74844717	0,678670254	0,039808831	0,238852983

Supplementary Table 3. Results from the cox proportional hazard analysis in relation to 5-year survival.

"rs_11pa" : rissk-score from the model
"age": individual age at time of diagnose
"bmi" : BMI at time of diagnosis
"stage" : Tumour stage (I-IV) at time of diagnosis.
"clinCA125": Clinically measured MUCIN-16 (CA-125) at time of diagnosis.
"q-value": Bonferroni (nr of tests in each sub-group) adjusted p-value.

Including all stages

	coef	exp(coef)	se(coef)	z	Pr(>|z|)	q-value
age	-0,00128918	0,998711651	0,019596071	-0,065787662	0,947546879	1
bmi	0,003804844	1,003812091	0,024283896	0,156681756	0,875495666	1
rs_11pa	4,70680448	110,6978569	1,98161825	2,375232707	0,017537889	0,087689447
stage	1,339859083	3,818505377	0,374645312	3,576340182	0,000348438	0,001742191
clinCA125	-3,09E-05	0,999969105	0,000103407	-0,298771298	0,765114552	1

Exluding stage information

	coef	exp(coef)	se(coef)	z	Pr(>|z|)	q-value
age	-0,004346378	0,995663054	0,018873909	-0,230284993	0,817870321	1
bmi	-0,010127262	0,989923846	0,03510039	-0,288522779	0,772946596	1
rs_11pa	6,265073938	525,8804672	2,025179457	3,093589517	0,001977509	0,007910035
clinCA125	-4,79E-06	0,999995214	0,000112818	-0,042423384	0,966161188	1

Restricted to stage III

	coef	exp(coef)	se(coef)	z	Pr(>|z|)	q-value
age	0,001257069	1,00125786	0,030452066	0,041280264	0,967072467	1
bmi	0,016600236	1,016738786	0,015395979	1,078218933	0,280936065	1
rs_11pa	7,079461316	1187,328751	3,532331461	2,004189412	0,045049774	0,180199097
clinCA125	-0,000327757	0,999672297	0,000260688	-1,257277792	0,208653063	0,834612252

Restricted to stage IV

	coef	exp(coef)	se(coef)	z	Pr(>|z|)	q-value
age	-0,01224158	0,987833043	0,026483791	-0,46222915	0,643917001	1
bmi	-0,208797696	0,811559401	0,074024079	-2,820672683	0,004792307	0,019169229
rs_11pa	3,223558834	25,1173498	2,875247974	1,121141155	0,262227783	1
clinCA125	6,49E-05	1,000064947	0,000106591	0,609284688	0,542335755	1
