# Supplementary material for: Data-driven analysis of a validated risk score for ovarian cancer identifies clinically distinct patterns during follow-up and treatment
Source: Commun Med (Lond). 2022 Oct 1;2:124. doi: 10.1038/s43856-022-00193-6 (PMC9526736; doi:10.1038/s43856-022-00193-6)
Supplement: Supplementary file 3 — Reporting Summary [file 43856_2022_193_MOESM3_ESM.pdf]

nature portfolio

Corresponding author(s): Stefan Enroth

Last updated by author(s): Nov 11, 2021

Reporting Summary

Nature Portfolio wishes to improve the reproducibility of the work that we publish. This form provides structure for consistency and transparency in reporting. For further information on Nature Portfolio policies, see our Editorial Policies and the Editorial Policy Checklist.

Statistics

For all statistical analyses, confirm that the following items are present in the figure legend, table legend, main text, or Methods section.

n/a	Confirmed	
[ ]	[x]	The exact sample size (*n*) for each experimental group/condition, given as a discrete number and unit of measurement
[ ]	[x]	A statement on whether measurements were taken from distinct samples or whether the same sample was measured repeatedly
[ ]	[x]	The statistical test(s) used AND whether they are one- or two-sided<br>*Only common tests should be described solely by name; describe more complex techniques in the Methods section.*
[x]	[ ]	A description of all covariates tested
[x]	[ ]	A description of any assumptions or corrections, such as tests of normality and adjustment for multiple comparisons
[ ]	[x]	A full description of the statistical parameters including central tendency (e.g. means) or other basic estimates (e.g. regression coefficient) AND variation (e.g. standard deviation) or associated estimates of uncertainty (e.g. confidence intervals)
[ ]	[x]	For null hypothesis testing, the test statistic (e.g. *F*, *t*, *r*) with confidence intervals, effect sizes, degrees of freedom and *P* value noted<br>*Give P values as exact values whenever suitable.*
[x]	[ ]	For Bayesian analysis, information on the choice of priors and Markov chain Monte Carlo settings
[x]	[ ]	For hierarchical and complex designs, identification of the appropriate level for tests and full reporting of outcomes
[x]	[ ]	Estimates of effect sizes (e.g. Cohen's *d*, Pearson's *r*), indicating how they were calculated

Our web collection on statistics for biologists contains articles on many of the points above.

Software and code

Policy information about availability of computer code

Data collection	No special software was used for data collection.
Data analysis	R version 4.0.3 was used to analyze the data.

For manuscripts utilizing custom algorithms or software that are central to the research but not yet described in published literature, software must be made available to editors and reviewers. We strongly encourage code deposition in a community repository (e.g. GitHub). See the Nature Portfolio guidelines for submitting code & software for further information.

Data

Policy information about availability of data

All manuscripts must include a data availability statement. This statement should provide the following information, where applicable:
- Accession codes, unique identifiers, or web links for publicly available datasets
- A description of any restrictions on data availability
- For clinical datasets or third party data, please ensure that the statement adheres to our policy

The datasets generated during the current study are available from the authors on reasonable request.

Field-specific reporting

Life sciences study design

All studies must disclose on these points even when the disclosure is negative.

Sample size	No sample size calculation was done. All samples fulfilling the criteria on diagnoses etc available in the participating clinical biobanks were used.
Data exclusions	Initial QC on available sample (e.g. plasma) amount available excluded some samples completely from the molecular analyses. Additional exclusion criteria (QC) were applied on the generated data and identified additional samples that was excluded. This is described in detail in Methods section.
Replication	This study has two parts, the first being to replicate/validate the performance of the risk-score developed in a previous study and the second to investigate how the risk-score develops after treatment. The first part confirmed the performance of the risk-score from the previous study. This second part is partly self-replicating since a decrease in the risk-score was seen in relation to clinically successful treatment. It is however not replicated using a second cohort as we did not have access to additional samples collecting during a similar time-period post-treatment.
Randomization	This is not relevant to the current study. Samples were divided based on clinical diagnoses and the performance of the risk-score was evaluated in these groups. In the second part, however, a unsupervised data-driven clustering based on the risk-score identified clinically valid subgroups.
Blinding	Blinding was not relevant to the study. The samples were collected in clinical context and analyzed based on diagnoses.

Reporting for specific materials, systems and methods

We require information from authors about some types of materials, experimental systems and methods used in many studies. Here, indicate whether each material, system or method listed is relevant to your study. If you are not sure if a list item applies to your research, read the appropriate section before selecting a response.

Materials & experimental systems

n/a	Involved in the study	
☒	☐	Antibodies
☒	☐	Eukaryotic cell lines
☒	☐	Palaeontology and archaeology
☒	☐	Animals and other organisms
☐	☒	Human research participants
☒	☐	Clinical data
☒	☐	Dual use research of concern

Methods

n/a	Involved in the study	
☒	☐	ChIP-seq
☒	☐	Flow cytometry
☒	☐	MRI-based neuroimaging

Human research participants

Policy information about studies involving human research participants

Population characteristics	Women from three cohorts were used. Two clinical cohorts with patients with suspected ovarian cancer, later surgically diagnosed with either benign or malign tumors. Several clinical variables including age, past and current diagnosis, treatment regime and survival data was available. No genetic information was available. The third cohort is a cross-sectional cohort with a large amount of information available including genetic information. Here, only sex and age was used.
Recruitment	Participants from the first clinical cohort (Biomovca) was recruited at health-care centers as described in Lycke et al Gynecologic Oncology 151 (2018) 159–165. Patients in the second clinical cohort is part of a large biobank, U-CAN, which collects samples from all consenting cancer patients treated at the Uppsala University Hospital, Sweden (Glimelius et al, ACTA ONCOLOGICA, 2018 VOL. 57 NO 2. 187 - 194). Participants in the cross-sectional cohort (NSPHS) was invited to participate based on geographical location (Igl et al, Rural and Remote Health, 2010, 11: 1363).<br><br>The analyzed samples from the third cohort was selected to be sex and age-matched with the distributions of the two clinical cohorts. No biases in recruitment with likely impact on the results have been identified.
Ethics oversight	Biomovca (Gothenburg University, Ref 139-13), U-CAN (Regionala Etikprövningsnämnden, Uppsala, Dnr: 2016/145) and NSPHS (Regionala Etikprövningsnämnden, Uppsala, Dnr. 2005:325 with approval of extended project period on 2016-03-19).

Note that full information on the approval of the study protocol must also be provided in the manuscript.
