# Supplementary material for: Data-driven analysis of a validated risk score for ovarian cancer identifies clinically distinct patterns during follow-up and treatment
Source: Commun Med (Lond). 2022 Oct 1;2:124. doi: 10.1038/s43856-022-00193-6 (PMC9526736; doi:10.1038/s43856-022-00193-6)
Supplement: Supplementary file 4 — Description of Additional Supplementary Files [file 43856_2022_193_MOESM4_ESM.pdf]

Description of Additional Supplementary Files

File name: Supplementary Data 1

Description: Source data for all figures presented in the manuscript. Data is presented as separate sheets, one for each figure, in a MS Excel file.
